# Influence of phylogenetic diversity of plant communities on tri-trophic interactions

**DOI:** 10.1007/s00442-023-05455-1

**Published:** 2023-09-30

**Authors:** Verónica Alavez, Rocio Santos-Gally, Manuel Gutiérrez-Aguilar, Ek del-Val, Karina Boege

**Affiliations:** 1 Instituto de Ecología, Departamento de Ecología Evolutiva, Universidad Nacional Autónoma de México C.P. 04510, Mexico City, Mexico; 2 Posgrado en Ciencias Biológicas, Universidad Nacional Autónoma de México, 04510 Mexico City, Mexico; 3 CONAHCYT-Instituto de Ecología, Departamento de Ecología Evolutiva, Universidad Nacional Autónoma de México, 04510 Mexico City, Mexico; 4 Departamento de Bioquímica, Facultad de Química, Universidad Nacional Autónoma de México, 04510 Mexico City, Mexico; 5 Instituto de Investigaciones en Ecosistemas y Sustentabilidad, Universidad Nacional Autónoma de México, 58190 Morelia, Michoacán Mexico

**Keywords:** Growth, Defenses, Herbivory, Life history, Phylogenetic diversity, Resource allocation, Tropical trees

## Abstract

Phylogenetic diversity of plant communities can influence the interaction between plants, herbivores, and their natural enemies. Plant communities with phylogenetically distant species tend to present a wide variety of functional traits and ecological niches, which in turn can influence competitive interactions among plants as well as food and habitat quality for herbivores and their natural enemies. To assess some different mechanisms by which phylogenetic diversity of plant communities can influence herbivores and their natural enemies, we established 12 experimental plots of tropical trees with two treatments: high and low phylogenetic diversity. We measured plant growth and anti-herbivore defenses, herbivore foliar damage, and predator activity in seven species that were present in both treatments. We found significant differences in the expression of plant traits as a function of species identity and their life history, but also depending on the phylogenetic context in which they grew. Pioneer species had higher growth and produced more phenolics in plots with high phylogenetic diversity versus plants in plots with low phylogenetic diversity. Accordingly, herbivore damage in these species was greater in plots with low phylogenetic diversity. Finally, predator activity on caterpillar clay models placed on plants was greater within the low phylogenetic diversity treatment, but only for non-myrmecophytic species. These results suggest that plant phylogenetic diversity can influence the expression of growth and defensive traits and further modify the interaction between plants, herbivores, and their natural enemies. However, such effects depend on plant life history and the presence of mutualistic interaction with ants.

**Supplementary Information:**

The online version contains supplementary material available at 10.1007/s00442-023-05455-1.

## Introduction

Phylogenetic diversity of plant communities has the potential to change the interaction between plants and their herbivores. Specifically, plant communities with high phylogenetic diversity have distantly related species with a wide diversity of functional traits, which can directly influence both herbivores and their natural enemies (Dinnage et al. 2012; Srivastava et al. 2012). One of the mechanisms by which these effects can occur is through changes in resource availability and different trade-offs between growth and defense against herbivores (Ballaré and Austin 2019), as well as through changes in the vegetation structure influencing the activity of the third trophic level (Staab et al. 2016, 2021).

The evolutionary relationships among species that make up a community can be visualized under the concept of phylogenetic diversity (Faith 1992), which can determine the ecological, phenotypic, and functional dissimilarity within communities, and therefore allows the distinction of different available ecological niches and species assembly across different guilds and trophic levels (Flynn et al. 2011). Communities with phylogenetically distant groups are composed of species with diverse evolutionary histories with different functional traits among species. In these communities, competition can be assumed to be low due to different resource-use among nonrelated species. In contrast, communities of evolutionary-related species with low phylogenetic diversity tend to present lower functional variation (Díaz et al. 2013) and hence similar ecological niches and resource-use strategies, therefore increasing the intensity of interspecific competition.

Previous studies have suggested that phylogenetic diverse neighborhoods can affect multiple features of ecosystems, such as productivity (Hao et al. 2018; Mazzochini et al. 2019), ecosystem stability (Cadotte et al. 2012), community resistance to drought (Chaves et al. 2021), and diversity of higher trophic levels (Dinnage et al. 2012; Staab et al. 2021). Phylogenetic diversity can also affect the intensity of species interactions, such as competition and herbivory, influencing the adaptive value and priorities of plant responses to these biotic stressors. For example, a positive relationship between plant phylogenetic diversity and the number of herbivore species has been reported (Dinnage et al. 2012; Lind et al. 2015; Wang et al. 2019, 2020). The resulting herbivore damage, however, should be influenced by herbivore´s diet breath and their abilities of host recognition, as a function of the diversity of defensive traits and structural complexity in a given plant community (Plath et al. 2012). This, in turn, should promote different associational effects, as described next.

Associational resistance occurs when plant neighborhood reduces the probability of one focal plant being found and consumed by herbivores, due to the reduced chemical or physical apparency of focal plants and/or the enhanced activity of natural enemies. On the contrary, associational susceptibility occurs when plants with palatable and/or similar traits grow in proximity, offering additional food sources for herbivores (Barbosa et al. 2009). In this context, associational susceptibility may be common in plant communities with low phylogenetic diversity and closely related species, leading to increased host recognition and consumption by specialist herbivores due to a high likelihood of plants sharing some functional traits (i.e., the same type of defenses; Castagneyrol et al. 2013). In contrast, plant communities with high phylogenetic diversity should experience greater associational resistance against specialist herbivores, owing the diversity of host plants with different defensive strategies and increased activity at the third trophic level (Ødegaard et al. 2005; Jactel et al. 2021). These mechanisms have been proven in agricultural contexts where push–pull systems provide effective means to protect crops from herbivores (Kebede et al. 2018); however, no empirical evidence of this is available for tropical forests.

An unexplored mechanism under which phylogenetic relationships among neighboring plants can influence plant–herbivore interactions is through the relative fitness costs of competition and herbivory, which lead to plant responses driven by growth and defense trade-offs. These trade-offs have been explained by different hypotheses and theories (Stamp 2003), mostly considering substrate-driven resource allocation costs (Monson et al. 2022) and natural selection on growth and defensive traits under various environmental scenarios (Coley et al. 1985; Ballaré and Austin 2019). For example, the growth–differentiation balance hypothesis considers source–sink activities to predict how the activity of primary and secondary metabolisms can be optimized (Herms and Mattson 1992). Likewise, the resource allocation hypothesis is based on the adaptive value of increasing plant defense when resources are limited for growth, considering the fitness costs for the replacement of tissues lost by herbivore damage (Coley et al. 1985). Novel approaches have highlighted the mechanistic and metabolic processes behind the regulation of growth and defense as an integral adaptive process responding to simultaneous biotic (e.g., competition and herbivory) and abiotic (e.g., resource availability) stressors (Ballaré and Austin 2019). For example, Monson et al. (2022) propose that the existence of stored resource reserves provides safety margins for biotic and abiotic stress-associated responses, which regulates the expression of plant responses to competition and herbivory, through different cross-talk pathways and transcriptional signal cascades. Within these cascades, the jasmonate pathway can influence primary and specialized metabolisms through the control of repressor–transcription factor complexes, regulating shade avoidance responses to competitors and defense strategies to deter herbivores (Guo et al. 2018; Ballaré and Austin 2019). Therefore, the optimization of plant responses involving growth and defense is now considered the result of a set of biochemical, developmental, configurational, physiological, and phenotypic trade-offs (Ballaré and Austin 2019; Monson et al. 2022), based on the fitness costs and the benefits of expressing growth and defense traits in particular contexts. Notably, resource availability can influence such trade-offs at different levels, promoting metabolic homeostasis (Monson et al. 2022), and triggering plant responses to avoid competition and respond to the selective pressure of herbivores (Ballaré and Austin 2019).

Assuming that plant phylogenetic diversity is positively related to niche diversification resulting in low competition for resources (Valiente-Banuet and Verdú 2007; Verdú et al. 2012; Navarro-Cano et al. 2016), we could expect reduced interspecific competition in communities with high phylogenetic diversity and hence, reduced growth-related trade-offs to respond to herbivore attacks. On the contrary, if competition is strong enough to trigger shade avoidance responses in plant communities with low phylogenetic diversity, a reduction in the production of defensive traits could be expected (Ballaré and Austin 2019). However, the influence of herbivore pressure due to associational susceptibility might represent a greater fitness challenge than competing with neighbors in these communities, favoring the production of defensive traits over plant growth.

Because changes in resource allocation are often context-dependent (Cipollini et al. 2014) and vary as a function of plant life history (Coley 1983; Salguero-Gómez et al. 2016), different strategies are expressed to optimize fitness, promoting trade-offs in the investment of resources for the expression of growth and defense traits (Lundgren and Marais 2020). For example, fast-growing plants, evolved in environments with high resource availability, are characterized by short-lived leaves and low levels of defenses against herbivores, while slow-growing species are adapted to more stressful, resource-limited conditions and produce long-lived leaves and a higher allocation of resources to defenses (Coley 1983; Adler et al. 2014). Thus, trade-offs related to resource limitation are expected to be more evident in fast-growing species, as their adaptive strategies to deal with both competitors and herbivores predominantly relay on plant growth and high resource availability (Coley et al. 1985).

The activity of predators is an additional factor playing a crucial role in plant–herbivore interactions. An increase in phylogenetic diversity of plant communities could influence predator activity, through a variety of plant functional plant traits (Dinnage et al. 2012; Schuldt et al. 2014). Specifically, in plant communities with high phylogenetic diversity, parasitoid abundance and parasitism rates have been found to be high, given the diversity of refuges, microclimates, and volatile compounds that directly affect the activity of parasitoids (Salazar et al. 2016; Staab et al. 2016). Regarding predation by ants, previous studies have reported that plant species richness increases their activity as predators in tropical forests (Leles et al. 2017). In the case of ants depending on myrmecophilous plant rewards, plant diversity driven by the abiotic environment can influence the composition of their communities (Rico-Gray 1993; Dáttilo et al. 2013a). Moreover, the spatial aggregation and the architecture of plants can be particularly relevant for their foraging patterns (Dáttilo et al. 2013b), given their low mobility and dependence on extrafloral nectaries and food bodies (Dáttilo et al. 2013a, b). Last, the quality of food rewards and domatia can be modified as a function of resources available for myrmecophilous plants (Heil et al 2001). Hence, if plant phylogenetic diversity modifies resource availability, canopy structural complexity, and the diversity of microclimates, ant–plant interactions should be particularly sensitive to changes in vegetation species composition. Overall, changes in the activity of all predator guilds can directly affect herbivore density and indirectly influence the level of damage they cause to plants (Sipura 1999; Van Bael et al. 2003). However, to our knowledge, there is no explicit empirical evidence available on the role of plant phylogenetic diversity on the activity of the third trophic level on herbivores.

In this context, the aim of this investigation was to assess the interactions between plants, herbivores, and natural enemies in experimental tropical tree communities with contrasting levels of phylogenetic diversity. We hypothesized that the diversity of niches and the strategies for resource acquisition associated with phylogenetic diversity should promote increased competition and lower structural complexity among plants with close evolutionary histories in communities with low phylogenetic diversity. Hence, we expected plants in communities with high phylogenetic diversity to exhibit greater growth, lower defense, and increased activity of the third trophic level compared to plants in communities with low phylogenetic diversity. Furthermore, we predicted that plants with fewer defenses in communities with high phylogenetic diversity should experience greater damage than plants in communities with low phylogenetic diversity. However, if associational resistance drives herbivore communities, this pattern should be reversed.

## Methods

### Study site

We carried out the study at the cattle ranch “Los Amigos” (18°32ʹ56″ N 94°59′58″ W) located in the municipality of Catemaco, Veracruz, Mexico, within the Biosphere Reserve of Los Tuxtlas. Vegetation in this area is a tropical rain forest with warm humid weather, an average temperature of 24.1–27.2 °C and an annual rainfall ranging between 1272 and 4201 mm, with a partially dry season between March and May (Gutiérrez-García and Ricker 2011). The site is characterized by a landscape of forest fragments and induced grassland areas for cattle ranching. The soil properties of the area are characterized by an acid pH (< 5.5), high mean values of iron (Fe) (89.95 mg / kg) and Mg (4.83 meq / 100 g), intermediate levels of Ca (4.54 meq / 100 g) and low values of exchangeable basic cation for the available bases (Ca and Mg; 9.81 meq / 100 g), high levels of organic matter (6.5%), with intermediate levels of P (6.37 ppm), and 0.22% to 0.43% of total N and between 3.2 and 5.4% of total C (Santos-Gally et al., unpublished manuscript). It should be noted that the affinity of iron oxides and hydroxides for phosphorous is likely to promote a limitation on the availability of this resource for plants, particularly in highly acidic soils (Chacon et al. 2006; Bortoluzzi et al. 2015).

Between 2018 and 2019, we established 12 experimental 15 × 15 m plots within the grassland areas of the study site (Santos-Gally and Boege 2022), close to native vegetation that provided a source of native herbivores and natural predators (supplementary material S1). Seedlings from 43 tree species were planted in two different arrays or treatments, so that half of the plots had high phylogenetic diversity (HPD), with 28 species that were distantly related, and the other half had low phylogenetic diversity (LPD), with 28 species sharing more recent common ancestors (see below). Twelve species were shared between treatments. Each plot had between 1 and 7 individuals from each species (*N* = 196 individuals/plot), although some of them did not survive during transplantation, resulting in a range between 21 and 28 species per plot, but the final species arrays maintained the contrast in phylogenetic diversity between both treatments (Santos-Gally et al. unpublished manuscript).

To assess differences in the phylogenetic structure between HDP and LPD plots, we calculated the mean pairwise distance (MPD) for the species within each plot. This measure informs on how closely related an average pair of species in a community is. We then obtained the standardized index of such distance (SES.MPD), to be contrasted against 1000 null assemblages calculated from a subset of random species from the local regional species pool (Webb et al. 2002). The values of SES.MPD for groups of species within HDP plots were always positive (**≥ **1.8) and higher than those of the null assemblages, indicating communities over-dispersed in the phylogeny. In contrast, SES.MPD values of LPD plots were negative (> −4.5) and lower than those of the null assemblages, resulting from clustered communities. These indices were calculated using the ape (Paradis and Schliep 2019) and picante (Kembel et al. 2010) packages in R 3.3.0 (R Development Core Team 2016).

Although both treatments shared 12 species, only seven species had enough surviving individuals to be compared between treatments: *Vachellia cornigera*, *Cedrela odorata*, *Cecropia obtusifolia*, *Ceiba pentandra*, *Zanthoxylum limonella*, *Eugenia acapulcensis*, and *Inga vera*. The first four are pioneer gap-dependent fast-growing species and the rest are shade-tolerant species. Additionally, three of them have facultative mutualistic interactions with ants (*V. cornigera*,* C. obtusifolia*, and *I. vera*) (Supplementary material S3). For each focal species, 20 to 40 individuals (depending on the survival of the individuals in the 12 plots) were randomly chosen in both treatments, with a total of 248 individuals (116 in HPD and 132 in LPD). To assess whether plant–herbivore interactions differed between plant communities within both treatments, for each plant, we estimated growth, physical and chemical defenses, foliar damage caused by herbivores, and the activity of the third trophic level, as described below.

### Plant growth

To estimate plant growth of focal species, we measured plant height and stem diameter at the beginning of the experiment, in June 2019 and two years later, in June 2021. Relative growth for each attribute was calculated with the formula:

$${\text{Relative}}\,{\text{growth}}\, = \,\frac{{X_{t1} - X_{t0} }}{{X_{t0} }}$$

where X represents each growth attribute, *t*_0_ corresponds to the initial measurements in 2019 and *t*_1_ corresponds to the measurements in 2021 for each growth trait.

### Defenses

To assess physical defenses, three mature leaves from each focal individual were collected in 2021 and used to measure leaf thickness and leaf mass per area (LMA = leaf dry mass/ leaf area) as indirect measures of leaf toughness (Onoda et al. 2011). Leaf thickness was measured using a digital micrometer, in the middle of the leaf (avoiding major veins). The leaf area was calculated with the Petiole application, and the dry weight was assessed using an analytical balance after drying the leaves at 60 °C for 48 h. The average values of these traits were calculated for each individual plant.

To estimate the production of phenolic compounds, in 2021, we collected three mature leaves from each plant, which were flash-frozen in liquid nitrogen, transported to the laboratory, and stored at −80 °C. Subsequently, the tissue was lyophilized and ground to a fine powder with a TissueLyser. Free phenolic acids were extracted following the improved microscale extraction protocol proposed by Zavala-López and Garcia-Lara (2017). Briefly, free phenolic acid extraction was performed by adding 0.7 ml of 80% methanol to 50 mg of leaf samples, mixed for 5 min in a vortex and incubated at 25° C for 10 min under constant stirring. The supernatant was then centrifuged for 10 min at 5000 rpm at 25 °C, decanted in a new Eppendorf tube, and stored at −20 °C until quantification. Free phenolic acids were assessed spectrophotometrically using the Folin–Ciocalteu reagent on a plate reader at 765 nm with a gallic acid standard curve (Zavala-López and Garca-Lara 2017).

### Leaf damage by herbivores

We calculated the average percentage of leaf damage by herbivores from the damage estimated in 5–12 leaves per individual, depending on the species and plant size. Leaves were collected in a systematic way covering the different strata of the individual. For this, we calculated the total number of leaves in the plant and divided this number by ten, and the resulting number *x* was used to collect every *x* leaf throughout the canopy. The percentage of leaf damage caused by chewing herbivores was estimated using the BioLeaf application for android systems (Machado et al. 2016). To quantify the percentage of leaf damage of the pinnately compound *V. cornigera* leaves, the percentage of absent pinnulas was counted in 10 leaflets randomly collected for each plant.

### Third trophic level activity

Predation rates were estimated using caterpillar models made with light green non-toxic modeling clay of 30 mm long by 3 mm wide, which has been shown to be efficient in determining predator activity (Roslin et al. 2017). A total of 563 caterpillars were placed on plants of the focal plant species, having 40–50 caterpillars in each plot and a total of 278 and 285 in the treatments of the high and low phylogenetic diversity, respectively. The models were glued to the leaf with KolaLoka^®^ glue and then checked after 24, 48, and 72 h. Models showing any damage were removed from the leaf (without replacement), photographed, and stored in wax paper bags for later identification of the type of attack (e.g., by birds, wasps, ants, etc.; Low et al. 2014).

### Statistical analysis

The values of relative height growth, relative diameter growth at the base of the stem, average leaf thickness, average specific leaf mass, total free phenol concentration, and percentage of leaf damage were transformed with the natural logarithm to satisfy the assumptions of normality and homoscedasticity and then analyzed with linear mixed model (LMM) using the 'lme' function of the nlme package.

The models to determine the effect of plant phylogenetic diversity treatments on each response variable considered as fixed explanatory variables—treatment, life history, and their interaction, and as random explanatory variables, the species nested in blocks. The blocks consisted of pairs of physically close plots (one of each treatment) to control variation in landscape attributes (Supplementary material S1). To assess the differences between treatments and life histories when the interaction term was significant in the LMM, we performed a contrast analysis using the 'lsmeans' function of the lsmeans package, in the model described above and generating the contrasts based on life history. Additionally, to determine treatment effects on each of the species individually, multiple LMMs were carried out taking each of the attributes as response variables, the treatment as a fixed explanatory variable, and the block as a random explanatory variable.

To determine the existence of trade-offs in resource allocation between growth and defense traits, multiple LMMs were carried out in which either of the defensive traits, physical (thickness and specific leaf mass), or chemical (total soluble phenols) was considered as response variable; and relative height growth, treatment, and their interaction were considered as explanatory variables, including species identity as a random variable. To evaluate the effect of defenses on leaf damage, we also performed an LMM, considering leaf damage as a response variable, defenses, treatment and their interaction as explanatory variables, and species identity as a random variable.

The number of damaged and undamaged clay models was quantified in all plants of each species in each plot, regardless of the time elapsed, to further calculate the percent of damaged models per species in both treatments. A generalized linear mixed-effects model with Gamma distribution was fitted with the 'glmer' function of the lme4 package, considering the percent damaged clay models as response variable, as fixed explanatory variables the treatment, the presence/absence of myrmecophily and their interaction, and species as a random explanatory variable. The number of caterpillars damaged by vertebrates or invertebrates was compared with a chi-square test. All analyses were performed in R v.4.2.0 (R Core Team, 2022).

## Results

**Fig. 1:**
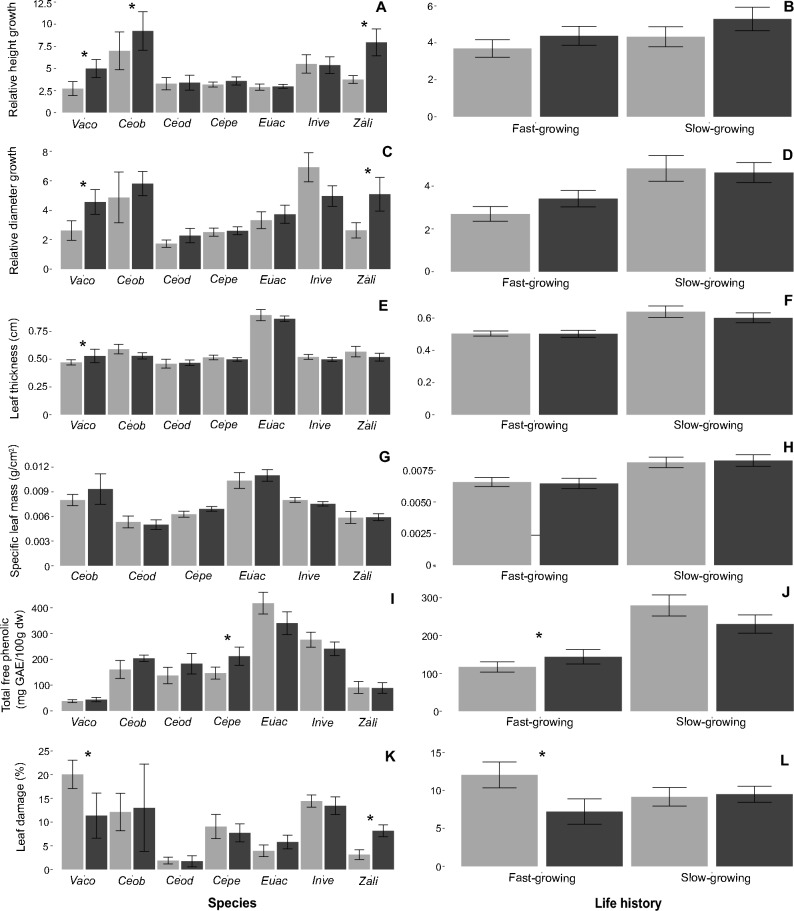
Growth in relative height (**A**, **B**) growth in relative diameter (**C**, **D**) leaf thickness (**E**, **F**) specific leaf mass (**G**, **H**) total free phenols (I, J) and leaf damage (**K**, **L**) in all focal species in plots with low (gray bars) and high (black bars) phylogenetic diversity. Plants were distinguished by their life history (pioneers and tolerant) and species identity: *Vachellia cornigera* (Vaco), *Cecropia obtusifolia* (Ceob), *Cedrela odorata* (Ceod), *Ceiba pentandra* (Cepe), *Eugenia acapulcensis* (Euac), *Inga vera* (Inve), and *Zanthoxylum limonella* (Zali). Mean and error bars (± 1 SE) are shown. Asterisks (*) indicate significant differences at < 0.05

We found a tendency of increased height of focal species in plots with high phylogenetic diversity in contrast with plants growing under low phylogenetic diversity (Fig. 1A), although the difference was not significant when sorting species by life history (pioneer or shade-tolerant; Fig. 1B). However, this effect was significant for plants of *V. cornigera* and *Z. limoncellum*, which doubled their height (*t* = −2.38, *p* = 0.02 and *t* = −2. 43, *p* = 0.03 respectively) and for *C. obtusifolia* plants, which grew 1.3 times more in HPD treatment compared to the LPD treatment (*t* = 4.57, *P* = 0.004) (Fig. 1A). The same pattern was found in the relative increase in diameter for most species, although only for *V. cornigera* and *Z. limoncellum*, such differences were significant, with a two-fold magnitude difference between treatments (*V. cornigera*: *t* = −2.17, *P* = 0.04; *Z. limoncellum*: *t* = −2.41, *P* = 0.03; Fig. 1C).

In the case of physical defenses, we observed that leaf thickness was different when considering the interaction between treatment and plant life history (*t* = −2.17, *P* = 0.03; Fig. 1F), where pioneer species had smaller differences between treatments than tolerant species. When analyzing the treatment effect for each species, we observed that the pioneer species *V. cornigera* had 1.12 times thicker leaves in the HPD treatment (*t* = −2.08, *P* = 0.05; Fig. 1E). For specific leaf mass, we found that it was 1.3 times greater in tolerant species than in pioneer than in shade-tolerant species (*t* = −2.46, *P* = 0.02; Fig. 1H), although we did not observe treatment effects for any life history or individual species (Fig. 1G).

**Fig. 2:**
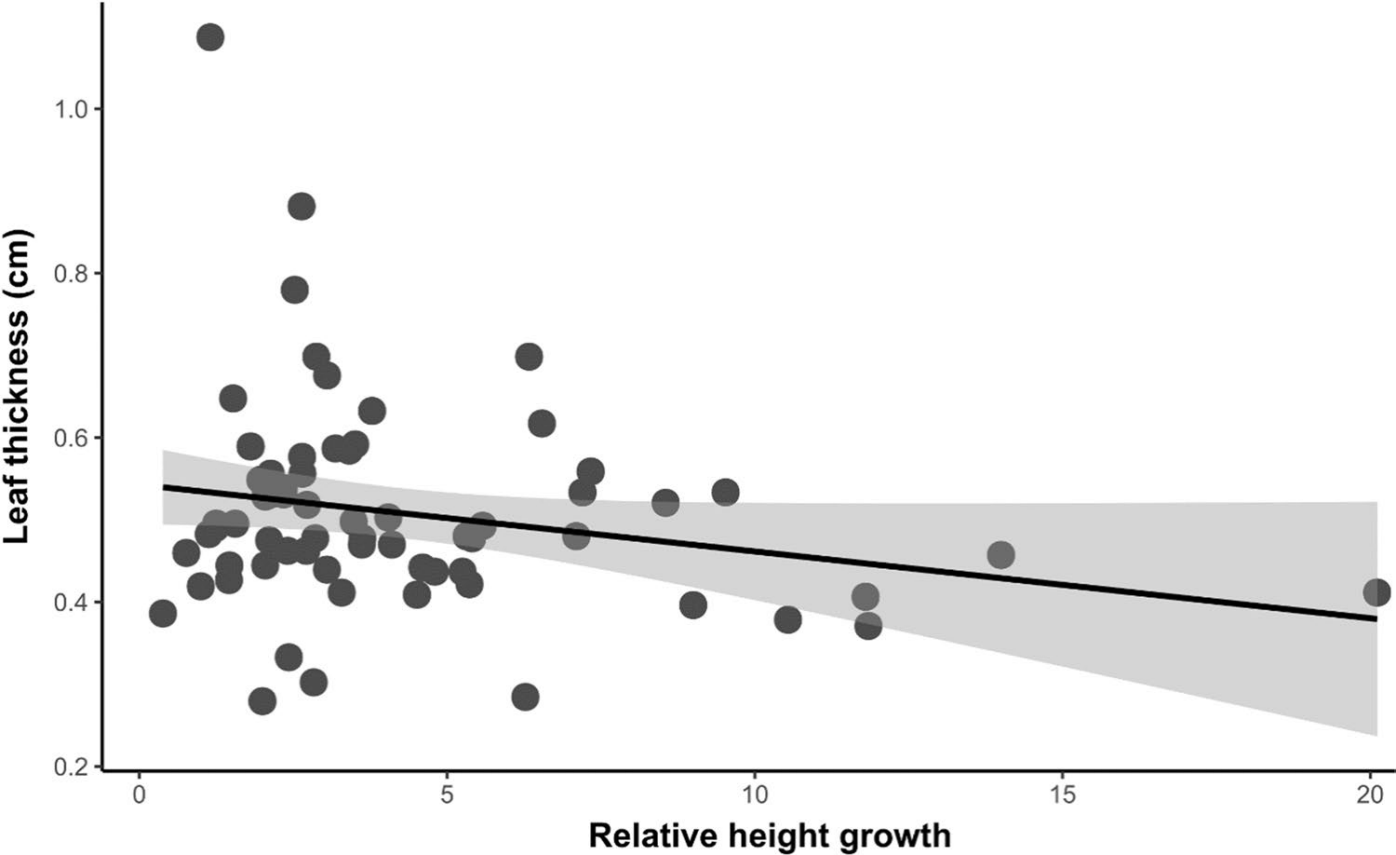
Relationships between relative height growth and leaf thickness in pioneer species. Black dots correspond to plants in the high phylogenetic diversity treatment and gray dots to plants in the low phylogenetic diversity treatment. The 95% confidence interval is indicated by the shaded area in gray

For total soluble phenols, we observed that there were differences between treatments, although varied as a function of the life history of plants (*t* = −2.08, *P* = 0.04). We found an increase of 19% in the concentration of total free phenols in HPD plots (*t* = 2.19, *P* = 0.03) only in pioneer species. Interestingly, the trend, although not significant, was the opposite pattern for tolerant species (Fig. 1J). By analyzing each species individually, we observed that *C. pentandra* showed a concentration of total soluble phenols 1.44 times higher in HPD than in LPD plots (*t* = −2.27, *P* = 0.03;Fig. 1I). When exploring trade-offs between resource allocation to growth and defense, we found a negative relationship between relative height growth and leaf thickness in pioneer species (*t* = −2.31, *P* = 0.02; Fig. 2), while the rest of the defenses had no relationship with growth traits for either pioneer or tolerant species, nor in any of the treatments.

**Fig. 3:**
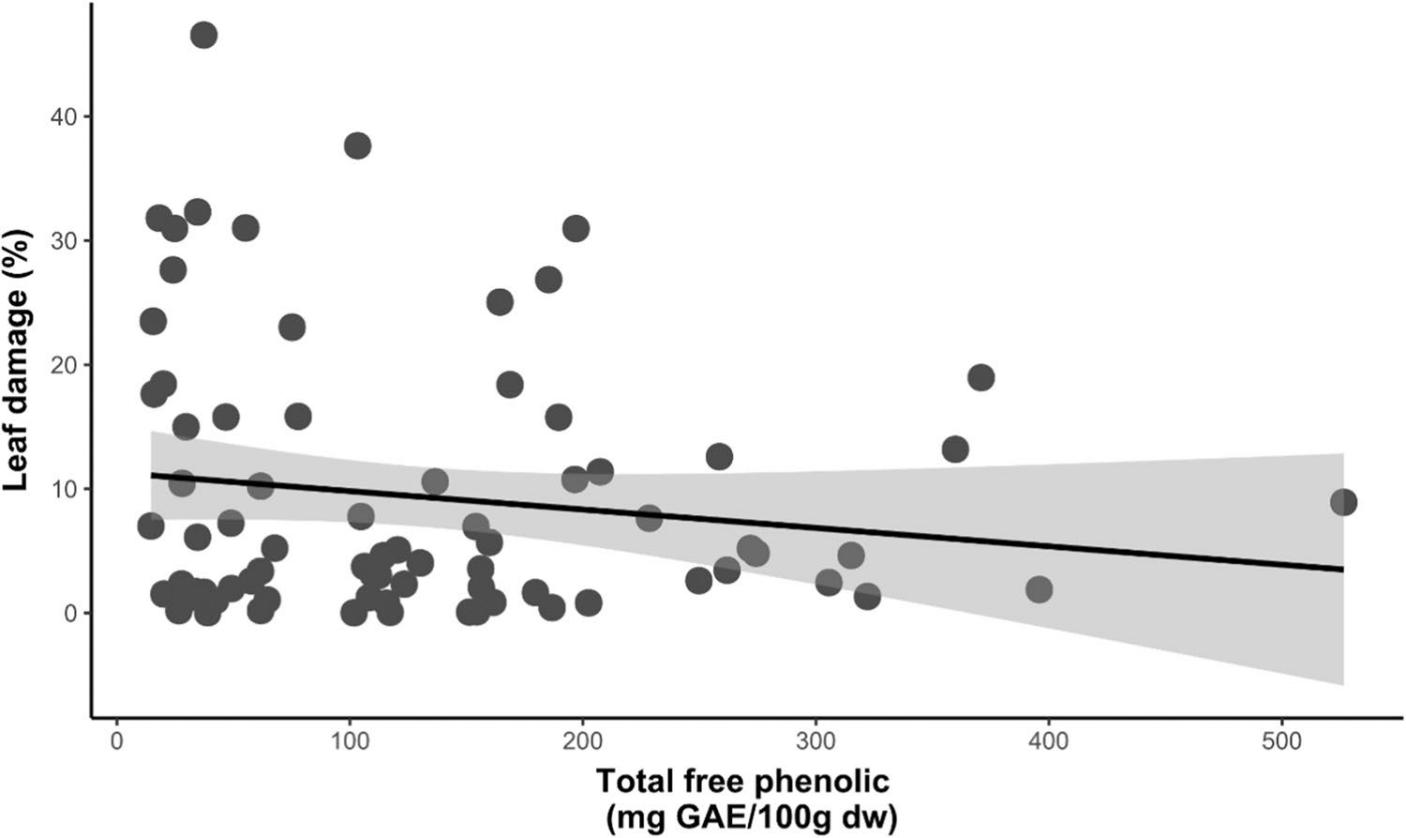
Relationships between total free phenolics and herbivory in pioneer species. Black dots correspond to plants in the high phylogenetic diversity treatment and gray dots to plants in the low phylogenetic diversity treatment. The 95% confidence interval is in indicated in y the shaded area in gray

**Fig. 4:**
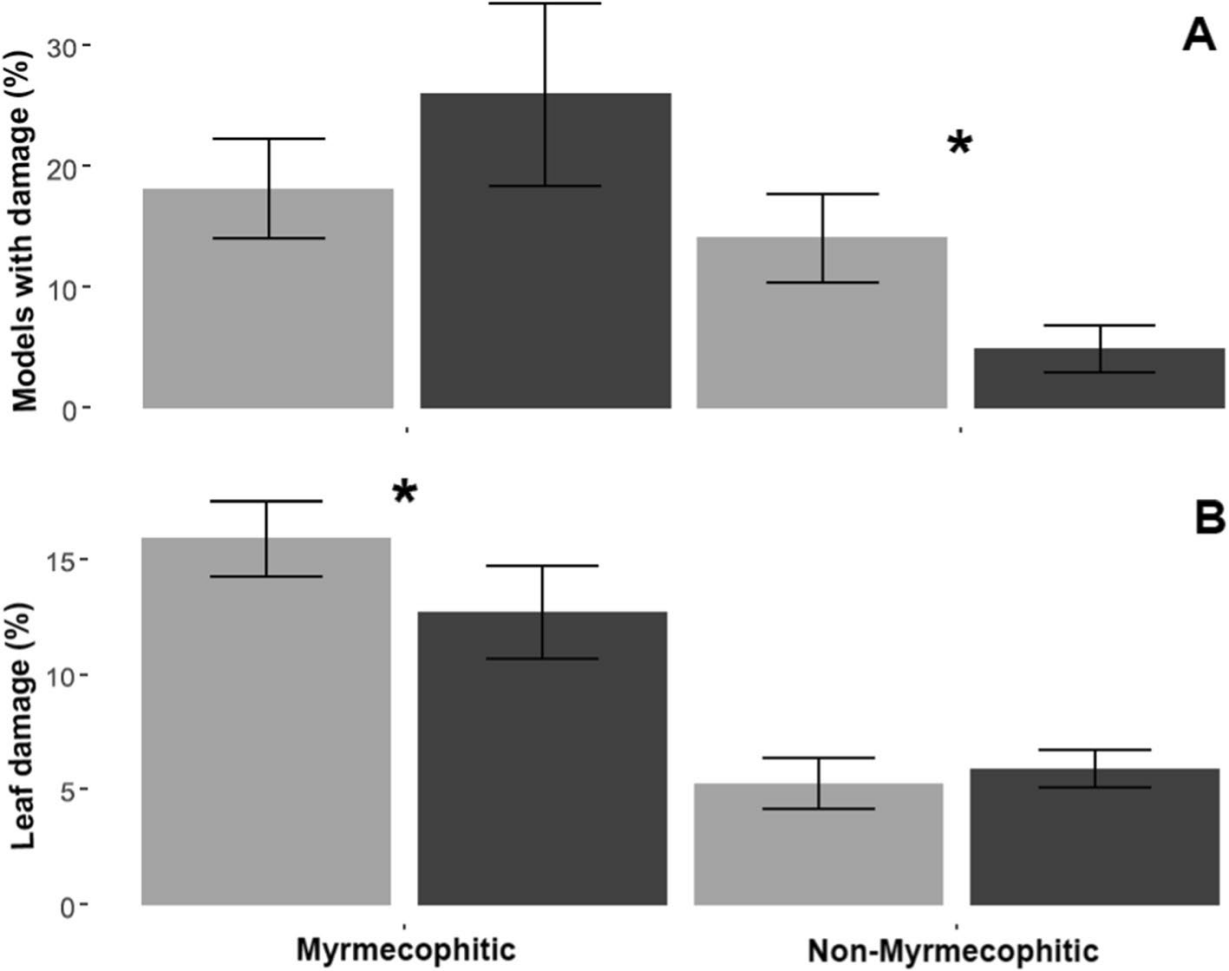
Response of the third trophic level (**A**) and the percentage of leaf damage (**B**) in myrmecophyte and non-myrmecophyte species in plots with high (black bars) and low (gray bars) phylogenetic diversity. Mean and error bars (± 1 SE) are shown. Asterisks (*) indicate significant differences at < 0.05

The percentage of leaf damage by herbivores was different between treatments according to the life history of plant species (*t* = 2.196, *P* = 0.03). Pioneer species had 1.7 times more damage in the LPD treatment compared to the HPD treatment (*t* = −2.61, *P* = 0.01). Furthermore, in this group of species, we found that the percentage of damage was negatively related to the concentration of total soluble phenols (*t* = 2.26, *P* = 0.03; Fig. 3). On the contrary, the tolerant species did not show differences between treatments (Fig. 1L). When analyzing the pattern for each species, we observed statistically significant differences for *V. cornigera*, which showed 8.7% less leaf damage in the HPD treatment than in the LPD treatment (*t* = 2.78, *P* = 0.012). In contrast, the tolerant species *Z. limoncellum* had 5.03% less leaf damage in LPD than in HPD plots (*t* = −2.34, *P* = 0.03; Fig. 1K). Furthermore, we observed that the percentage of leaf damage was different between treatments, but as a function of whether the species had mutualistic interactions with ants or not (*t* = −2.23, *P* = 0.027). In myrmecophytic species, there was 1.2 times greater leaf damage in LPD compared to HPD (*t* = −2.09, *P* = 0.04; Fig. 4B).

Regarding the activity of the third trophic level, the percentage of damaged caterpillars was explained by the interaction between treatment and the presence or absence of mutualistic ants (*t* = −2.22, *P* = 0.03; Fig. 4A). For non-myrmecophytic species, the percentage of damaged caterpillars was 2.5 times greater in LPD than in HPD plots (*t* = −1.97, *P* = 0.05). Interestingly, mutualistic species showed the opposite trend, although not significant, with greater number of caterpillars damaged in HPD than in LPD plots (*t* = 0.76, *P* = 0.44). In general, damage to more than 90% of caterpillar models was caused by invertebrates (χ^2^ = 69.57, *P* < 0.0001).

## Discussion

We report differences in plant growth and the expression of defensive traits within experimental plant communities with contrasting levels of plant phylogenetic diversity, although such variation was influenced by plant life history and species identity. Specifically, pioneer species had greater relative growth in height and diameter and increased amounts of defenses in HPD than LPD plots. Consequently, we also found that for these species, herbivore damage was greater in LPD than HPD plots. Finally, we report that predation by the third trophic level was greater in plots with LPD than plots with HPD, but only in non-myrmecophytic species. All these results together suggest that tri-trophic interactions among plants, herbivores, and their natural enemies can be influenced by the degree of the phylogenetic diversity of plant communities, but are contingent upon life history and species identity.

Although stem elongation is a common plant response to competition by neighboring plants in shade-intolerant species (Cipollini et al. 2014; Ballaré and Austin 2019), given the small canopies of saplings in our study, shading was unlikely to be the main driver of differences in plant growth between treatments. The increased growth in HDP plots appeared to be rather related to increased nutrient availability potentially resulting from greater niche partitioning in these plots according to our hypothesis, but could also be promoted by inequalities in the competitive ability of neighboring plant species (Chesson 2000; Emerson and Gillespie 2008). Hence, assessing changes in resource availability in our experimental plots warrants further investigation. Not sharing closely related common ancestors, species in HDP communities tend to have different morphological and functional attributes that allow them to make differential use of resources (Kraft et al. 2007; Cavender-Bares et al. 2009), leading to increased survival (Chaves et al. 2021), plant growth, and positive plant–plant interactions (Tucker et al. 2018; Teixeira et al. 2022). For example, some studies have observed that nurse species facilitate growth for distantly related species than for closely related ones (i.e., with high phylogenetic distance; Valiente-Banuet and Verdú, 2007). Accordingly, the lower growth rates observed for at least three of the studied species in plots with LPD could be related to overlapping niches with neighboring plants, promoting greater interspecific competition and less resource availability. Alternatively, increased herbivore pressure in these plots could have favored plant responses to deal with herbivory, rather than responses to neighboring plants (Ballaré and Austin 2019), as discussed next.

Given the variety of trade-offs between growth and defense driven by substrate availability, source–sink activities, metabolic cross-talk, and fine-tuned regulatory processes to optimize plant responses to competition and defense in stressful environments (Ballaré and Austin 2019; Monson et al. 2022), we expected that in low phylogenetic diversity plots, resource-limited plants would induce responses to avoid competition, potentially constraining their ability to defend against herbivores (Monson et al. 2022). Nevertheless, if associational susceptibility increased herbivore damage to the point where fitness costs exceeded those caused by competition, plant growth should have been downregulated, leading to an expected greater expression of defenses (Ballaré and Austin 2019).

Interestingly, our results indicate that the expression of growth and defense traits varied as a function of plant life history and species identity, suggesting differences in the adaptive responses to regulate metabolic and allocation strategies to simultaneously deal with competitors and herbivores (Ballaré and Austin 2019). Whereas in HPD plots associational resistance was expected, in LPD plots, associational susceptibility should be common given shared defensive strategies and architecture among evolutionary-related species. Hence, in the latter scenario, a greater herbivore pressure on new tissue could favor the production of plant defenses and inhibit plant responses to competition (Ballaré and Austin 2019). Although growth was indeed reduced in LPD plots, only shade-tolerant plants showed a tendency to increase their defenses in these plots, likely due to the high costs of replacing tissue lost by herbivore damage and their intrinsic low growth rates (Coley et al. 1985). In contrast, pioneer species reduced their investment in plant defense in this treatment relative to HDP plots, where they exhibited increased growth. This suggests a lack of trade-offs between the expression of growth and phenolic compounds, and instead, an adaptive advantage of defending new tissue, particularly in vigorous plants (Price 1991). To better understand these responses, it would be relevant to determine the regulatory pathways influencing these plastic plant responses as a function of the identity of neighboring plants, and the resulting resource availability and associational effects.

Although we did not directly measure leaf toughness, leaf thickness can be used as a good indicator of structural resistance when comparing leaves of the same material (i.e., same species, Onoda et al. 2011). We found that this component of structural resistance also varied between treatments as a function of the life history of plants. Interestingly, when considering all pioneer species, we found a negative relationship between relative height growth and leaf thickness, a common trade-off resulting from biomass allocation to stem elongation and leaves (Wright et al. 2004). In the case of LMA, where Rosado-Sánchez et al. (2018) report a positive effect of species diversity on physical defenses, we did not find differences for this structural defensive trait between phylogenetic diversity treatments. It is likely that the expression of LMA was driven by the light environment, which was similar between treatments due to the small sapling canopies of neighboring plants (Rosado-Sánchez et al. 2018).

Previous studies have shown the influence of plant species diversity on plant–herbivore interactions (Moreira et al. 2014; Castagneyrol et al. 2018; Muiruri et al. 2019), proposing changes in defenses against herbivores due to trade-offs in resource allocation, variation in foliar traits, or associational effects in communities as potential explanatory mechanisms, However, conclusive results have remained elusive. For instance, in the case of the tropical shade-tolerant species *Swietenia macrophylla*, changes in both the diversity of genotypes and species in the neighborhood have been reported to have a positive effect on the production of chemical defenses, without any apparent relationship with plant growth (Moreira et al. 2014). In contrast, another study with the herb *Jacobaea vulgaris* within experimental communities found that the increase in the number of plant species in the neighborhood was negatively related to the concentration of pyrrolizidine alkaloids of focal species (Kostenko et al. 2017), highlighting that neighborhood identity can affect the expression of this defense. Despite these previous findings, our study stands as the first experimental evidence addressing the effects of phylogenetic diversity on growth rates and the expression of defenses in tropical trees. We report that this level of diversity may also have important consequences for the relationship between plants, herbivores, and their natural enemies by modifying growth and defense patterns against their consumers.

### Leaf damage by herbivores

The herbivore damage observed (9.78%) in our experimental saplings is comparable to the average damage (9.3%) reported for seedlings in the region of Los Tuxtlas, Veracruz (de la Cruz and Dirzo 1987). Likewise, the average leaf damage of pioneer seedlings (10.13%) and tolerant seedlings (9.34%) is consistent with data from a previous study in the same region (pioneers: 11.6%; tolerant: 9.5%; Ruiz-Guerra et al. 2010). The percentage of leaf damage from herbivores was twice as high as in the LPD plots than in the HPD plots for the pioneer species, while the tolerant species showed no difference between treatments. Accordingly, other studies have found that the presence of distantly related species decreases leaf damage caused by herbivores due to associational resistance (Jactel and Brockerhoff 2007; Dinnage 2013; Castagneyrol et al. 2014). In our case, this mechanism, together with an increase in phenolics in pioneer plants, is a possible explanation for the lower percentage of leaf damage observed for pioneer species in the HDP plots.

Communities composed of distantly related species offer a wide variety of resources and species groups with different defensive strategies, which decreases the probability of specialist herbivores finding their host plant, thereby reducing their damage to plants (Hambäck and Beckerman 2003; Barbosa et al. 2009). In contrast, in LPD plots, associational susceptibility could explain why focal pioneer species, being phylogenetically closer to the rest of the community with low investments in defenses (Russell and Louda 2005; Dinnage 2013), experienced a greater percent of leaf damage. Because associational effects are influenced by the degree of herbivore specialization (Castagneyrol et al. 2014), knowledge of herbivore´s diet breath in our experimental plots will be relevant in future studies to better understand these indirect effects.

Our results highlight the relevance of considering plant life histories to understand plant–herbivore interactions in different communities. Pioneer species tend to receive greater attack from herbivores in their juvenile stages than in later developmental stages and are more responsive to resource availability than shade-tolerant species, prioritizing induced responses such as compensatory growth or the production of induced chemical defenses after detecting herbivore damage (Coley et al. 1985; Dayrell et al. 2018). This might explain why only pioneer species within HPD showed a greater concentration of total phenolics and less herbivore damage compared to plants within LPD. In contrast, shade-tolerant species rely more on constitutive defenses than pioneer species and are less likely to respond on the short term to resource limitations (Coley 1983). Nevertheless, considering that the expression of defenses in woody species increases during plant ontogeny (Barton and Koricheva 2010), it is possible that longer-term plant responses to phylogenetic diversity treatments could still occur at later stages of development, which warrants further investigation.

### Third trophic level

Plant diversity has been observed to increase predation rates and the number and abundance of predatory ant species in tropical and subtropical forests (Staab et al. 2014; Schuldt and Staab 2015; Leles et al. 2017). In this study, we report that this may depend on the presence of defensive mutualisms. The percentage of attacks on caterpillar models in non-myrmecophyte species was greater in LPD than in HPD plots, while in myrmecophyte species, we found the opposite trend, although the difference was not significant. While increased activity of the third trophic level in communities with high diversity has been demonstrated for various groups including ants, birds, and parasitoids (Poch and Simonetti 2013; Staab et al. 2016), our study found that more than 90% of the clay models showed marks made by arthropods, indicating that they were likely the main predator guild in the experimental plots.

In the case of myrmecophyte species, it is likely that ants were primarily responsible for the damage to caterpillar models. Being affected by habitat heterogeneity (Díaz-Castelazo et al. 2020), ants were probably more active in communities with HPD (Staab et al. 2016, 2021). In contrast, for species without mutualistic interaction with ants, we observed greater activity of the third trophic level in LPD than in HPD plots. This pattern could be explained by the complexity of the vegetation promoted by plant phylogenetic diversity, which plays an important role in herbivore-predator interactions, as it largely determines the availability of refugia for herbivores (Riihimäki et al. 2006). For example, previous studies have reported that predators, such as spiders (Riihimäki et al. 2006), birds (Whelan 1989), and parasitoids (Gingras et al. 2002), face greater difficulty in recognizing their prey in sites with greater vegetation complexity and structure, leading to lower herbivore predation rates.

We acknowledge that our study was conducted in synthetic experimental communities and therefore, plant responses to community phylogenetic diversity may be more variable and complex in natural communities, influenced by different mechanisms simultaneously operating at different spatial scales (Mutshinda et al. 2009; Wiegand et al. 2017). Thus, the interpretation of our results is constrained to the chosen species and the specific communities experimentally assembled, given the species-specific responses we report. However, by experimentally controlling plant phylogenetic diversity of tropical tree species, we were able to provide valuable insights into potential mechanisms operating in highly diverse ecosystems such as tropical forests. These findings could be further explored with different settings and more species in natural communities, adding depth to our understanding on the evolutionary ecology of plant–herbivore interactions.

## Conclusions

Our results highlight that, depending on life history and species identity of plants, phylogenetic diversity of plant communities can influence tri-trophic interactions through changes in resource allocation to growth and defense. Nevertheless, further considering different plant ontogenetic stages, herbivores’ diet breath and the inclusion of more defenses should confirm whether growth–defense trade-offs and/or associational effects are the main mechanisms by which phylogenetic diversity influences plant–herbivore interactions. Furthermore, investigating the regulatory pathways that allow plants to induce plastic responses to optimize fitness through growth and defense as a function of their community context, seems promissory to link the fields of evolutionary ecology and cellular biology to better understand the mechanisms influencing plant phenotypes and their influence on ecological interactions in highly diverse ecosystems (Monson et al. 2022).

### Supplementary Information

Below is the link to the electronic supplementary material.

Supplementary file1 (PDF 255 KB)

## Data Availability

The data sets generated during the current study are available from the corresponding author on reasonable request.

## References

[CR1] Adler PB, Salguero-Gómez R, Compagnoni A, Hsu JS, Ray-Mukherjee J, Mbeau-Ache C, Franco M (2014). Functional traits explain variation in plant lifehistory strategies. Proc Nat Acad Sci.

[CR2] Ballaré CL, Austin AT (2019). Recalculating growth and defense strategies under competition: key roles of photoreceptors and jasmonates. J Exp Bot.

[CR3] Barbosa P, Hines J, Kaplan I, Martinson H, Szczepaniec A, Szendrei Z (2009). Associational resistance and associational susceptibility: having right or wrong neighbors. Annu Rev Ecol Evol Syst.

[CR4] Barton KE, Koricheva J (2010). The ontogeny of plant defense and herbivory: characterizing general patterns using meta-analysis. Am Nat.

[CR5] Bortoluzzi EC, Pérez CAS, Ardisson JD, Tiecher T, Caner L (2015). Occurrence of iron and aluminum sesquioxides and their implications for the P sorption in subtropical soils. App Clay Sci.

[CR6] Cadotte MW, Dinnange R, Tilman D (2012). Phylogenetic diversity promotes ecosystem stability. Ecology.

[CR7] Castagneyrol B, Giffard B, Péré C, Jactel H (2013). Plant apparency, an overlooked driver of associational resistance to insect herbivory. J Ecol.

[CR8] Castagneyrol B, Jactel H, Vacher C, Brockerhoff EG, Koricheva J (2014). Effects of plant phylogenetic diversity on herbivory depend on herbivore specialization. J Appl Ecol.

[CR9] Castagneyrol B, Jactel H, Moreira X (2018). Anti-herbivore defences and insect herbivory: Interactive effects of drought and tree neighbours. J Ecol.

[CR10] Cavender-Bares J, Kozak KH, Fine PVA, Kembel SW (2009). The merging of community ecology and phylogenetic biology. Ecol Lett.

[CR11] Chacon N, Silver WL, Dubinsky EA, Cusack DF (2006). Iron reduction and soil phosphorus solubilization in humid tropical forests soils: the roles of labile carbon pools and an electron shuttle compound. Biogeochemistry.

[CR12] Chaves R, Ferrandis P, Escudero A, Luzuriaga AL (2021). Diverse phylogenetic neighborhoods enhance community resistance to drought in experimental assemblages. Sci Rep.

[CR13] Chesson P (2000). Mechanisms of maintenance of species diversity. Annu Rev Ecol Syst.

[CR14] Cipollini D, Walters D, Voelckel C (2014). Costs of resistance in plants: from theory to evidence. Annual plant reviews.

[CR15] Coley PD (1983). Herbivory and defensive characteristics of tree species in a lowland tropical forest. Ecol Monogr.

[CR16] Coley PD, Bryant JP, Chapin FS (1985). Resource availability and plant antiherbivore defense. Science.

[CR17] Dáttilo W, Rico-Gray V, Rodrigues DJ, Izzo TJ (2013). Soil and vegetation features determine the nested pattern of ant–plant networks in a tropical rainforest. Ecol Entomol.

[CR18] Dáttilo W, Guimaraes PR, Izzo TJ (2013). Spatial structure of ant–plant mutualistic networks. Oikos.

[CR19] Dayrell RLC, Arruda AJ, Pierce S, Negreiros D, Meyer PB, Lambers H, Silveira FAO (2018). Ontogenetic shifts in plant ecological strategies. Funct Ecol.

[CR20] de la Cruz M, Dirzo R (1987). A Survey of the Standing Levels of Herbivory in Seedlings from a Mexican Rain Forest. Biotropica.

[CR21] Díaz S, Purvis A, Cornelissen JHC, Mace GM, Donoghue MJ, Ewers RM, Jordano P, Pearse WD (2013). Functional traits, the phylogeny of function, and ecosystem service vulnerability. Ecol Evol.

[CR22] Díaz-Castelazo C, Martínez-Adriano CA, Dáttilo W, Rico-Gray V (2020). Relative contribution of ecological and biological attributes in the fine-grain structure of ant-plant networks. PeerJ.

[CR23] Dinnage R (2013). Phylogenetic diversity of plants alters the effect of species richness on invertebrate herbivory. PeerJ.

[CR24] Dinnage R, Cadotte MW, Haddad NM, Crutsinger GM, Tilman D (2012). Diversity of plant evolutionary lineages promotes arthropod diversity. Ecol Lett.

[CR25] Emerson BC, Gillespie RG (2008). Phylogenetic analysis of community assembly and structure over space and time. Trends Ecol Evol.

[CR26] Faith DP (1992). Conservation evaluation and phylogenetic diversity. Biol Conserv.

[CR27] Flynn DFB, Mirotchnick N, Jain M, Palmer MI, Naeem S (2011). Functional and phylogenetic diversity as predictors of biodiversity- Ecosystem-function relationships. Ecology.

[CR28] Gingras D, Dutilleul P, Boivin G (2002). Modeling the impact of plant structure on host-finding behavior of parasitoids. Oecologia.

[CR29] Guo Q, Major IT, Howe GA (2018). Resolution of growth–defense conflict: mechanistic insights from jasmonate signaling. Curr Opin Plant Biol.

[CR30] Gutiérrez-García G, Ricker M (2011). Climate and climate change in the region of Los Tuxtlas (Veracruz, Mexico): A statistical analysis. Atmosfera.

[CR31] Hambäck PA, Beckerman AP (2003). Herbivory and plant resource competition: a review of two interacting interactions. Oikos.

[CR32] Hao MH, Zhang C, Zhao X, von Gadow K (2018). Functional and phylogenetic diversity determine woody productivity in a temperate forest. Ecol Evol.

[CR33] Heil M, Hilpert A, Fiala B, Linsenmair KE (2001). Nutrient availability and indirect (biotic) defence in a Malaysian ant-plant. Oecologia.

[CR34] Herms DA, Mattson WJ (1992). The dilemma of plants: to grow or defend. Q Rev Bio.

[CR35] Jactel H, Brockerhoff EG (2007). Tree diversity reduces herbivory by forest insects. Ecol Lett.

[CR36] Jactel H, Moreira X, Castagneyrol B (2021). Tree diversity and forest resistance to insect pests: patterns, mechanisms, and prospects. Annu Rev Entomol.

[CR37] Kebede Y, Baudron F, Bianchi F, Tittonell P (2018). Unpacking the push-pull system: Assessing the contribution of companion crops along a gradient of landscape complexity. Agric Ecosyst Environ.

[CR38] Kembel SW, Cowan PD, Helmus MR, Cornwell WK, Morlon H, Ackerly DD, Blomberg SP, Webb CO (2010). Picante: R tools for integrating phylogenies and ecology. Bioinformatics.

[CR39] Kostenko O, Mulder PPJ, Courbois M, Bezemer TM (2017). Effects of plant diversity on the concentration of secondary plant metabolites and the density of arthropods on focal plants in the field. J Ecol.

[CR40] Kraft NJB, Cornwell WK, Webb CO, Ackerly DD (2007). Trait evolution, community assembly, and the phylogenetic structure of ecological communities. Am Nat.

[CR41] Leles B, Xiao X, Pasion BO, Nakamura A, Tomlinson KW (2017). Does plant diversity increase top—down control of herbivorous insects in tropical forest ?. Oikos.

[CR42] Lind EM, Vincent JB, Weiblen GD, Cavender-Bares J, Borer ET (2015). Trophic phylogenetics: Evolutionary influences on body size, feeding, and species associations in grassland arthropods. Ecology.

[CR43] Low PA, Sam K, Mcarthur C, Posa MRC, Hochuli DF (2014). Determining predator identity from attack marks left in model caterpillars: guidelines for best practice. Entomol Exp Et Appl.

[CR100] Lundgren MR, Des Marais DL (2020). Life history variation as a model for understanding trade-offs in plant–environment interactions. Curr Biol.

[CR44] Machado BB, Orue JPM, Arruda MS, Santos CV, Sarath DS, Goncalves WN, Silva GG (2016). BioLeaf: a professional mobile application to measure foliar damage caused by insect herbivory. Comput Electron Agric.

[CR45] Mazzochini GG, Fonseca CR, Costa GC, Santos RM, Oliveira-Filho AT, Ganade G (2019). Plant phylogenetic diversity stabilizes large-scale ecosystem productivity. Glob Ecol Biogeogr.

[CR46] Monson RK, Trowbridge AM, Lindroth RL, Lerdau MT (2022). Coordinated resource allocation to plant growth-defense trade-offs. New Phytol.

[CR47] Moreira X, Abdala-Roberts L, Parra-Tabla V, Mooney KA (2014). Positive effects of plant genotypic and species diversity on anti-herbivore defenses in a tropical tree species. PLoS ONE.

[CR48] Muiruri EW, Barantal S, Iason GR, Salminen J, Perez-Fernandez E, Koricheva J (2019). Forest diversity effects on insect herbivores : do leaf traits matter ?. New Phytol.

[CR49] Mutshinda CM, O’Hara RB, Woiwod IP (2009). What drives community dynamics?. Proc r Soc b: Biol Sci.

[CR50] Navarro-Cano JA, Ferrer-Gallego PP, Laguna E, Ferrando I, Goberna M, Valiente-Banuet A, Verdú M (2016). Restoring phylogenetic diversity through facilitation. Restor Ecol.

[CR51] Ødegaard F, Diserud OH, Østbye K (2005). The importance of plant relatedness for host utilization among phytophagous insects. Ecol Lett.

[CR52] Onoda Y, Westoby M, Adler PB, Choong AMF, Clissold FJ, Cornelissen JHC, Díaz S (2011). Global patterns of leaf mechanical properties. Ecol Lett.

[CR53] Paradis E, Schliep K (2019). Ape 5.0: An environment for modern phylogenetics and evolutionary analyses in R. Bioinformatics.

[CR54] Plath M, Dorn S, Riedel J, Barrios H, Mody K (2012). Associational resistance and associational susceptibility: Specialist herbivores show contrasting responses to tree stand diversification. Oecologia.

[CR55] Poch TJ, Simonetti JA (2013). Insectivory in *Pinus*
*radiata* plantations with different degree of structural complexity. For Ecol Manag.

[CR56] Price PW (1991). The plant vigor hypothesis and herbivore attack. Oikos.

[CR57] Rico-Gray V (1993). Use of plant-derived food resources by ants in the dry tropical lowlands of coastal Veracruz, México. Biotropica.

[CR58] Riihimäki J, Vehviläinen H, Kaitaniemi P, Koricheva J (2006). Host tree architecture mediates the effect of predators on herbivore survival. Ecol Entomol.

[CR59] Rosado-Sánchez S, Parra-Tabla V, Betancur-Ancona D, Moreira X, Abdala-Roberts L (2018). Effects of tree species diversity on insect herbivory and leaf defences in *Cordia*
*dodecandra*. Ecol Entomol.

[CR60] Roslin T, Andrew NR, Asmus A, Barrio IC, Basset Y (2017). Higher predation risk for insect prey at low latitudes and elevations. Science.

[CR61] Ruiz-Guerra B, Guevara R, Mariano NA, Dirzo R (2010). Insect herbivory declines with forest fragmentation and covaries with plant regeneration mode: evidence from a Mexican tropical rain forest. Oikos.

[CR62] Russell FL, Louda SM (2005). Indirect interaction between two native thistles mediated by an invasive exotic floral herbivore. Oecologia.

[CR63] Salazar D, Jaramillo A, Marquis RJ (2016). The impact of plant chemical diversity on plant—herbivore interactions at the community level. Oecologia.

[CR64] Salguero-Gómez R, Jones OR, Blomberg SP, Hodgson DJ, Zuidema PA, De Kroon H (2016). Fast–slow continuum and reproductive strategies structure plant life-history variation worldwide. Proc Natl Acad Sci USA.

[CR65] Santos-Gally R, Boege K, Mongagnini F (2022). Biodiversity Islands: the role of native tree islands within silvopastoral systems in a neotropical region. Biodiversity Islands: strategies for conservation in human-dominated environments.

[CR66] Schuldt A, Staab M (2015). Tree species richness strengthens relationships between ants and the functional composition of spider assemblages in a highly diverse forest. Biotropica.

[CR67] Schuldt A, Baruffol M, Bruelheide H, Chen S, Chi X, Wall M, Assmann T (2014). Woody plant phylogenetic diversity mediates bottom-up control of arthropod biomass in species-rich forests. Oecologia.

[CR68] Sipura M (1999). Tritrophic interactions: willows, herbivorous insects and insectivorous birds. Oecologia.

[CR69] Srivastava DS, Cadotte MW, Macdonald AAM, Marushia RG, Mirotchnick N (2012). Phylogenetic diversity and the functioning of ecosystems. Ecol Lett.

[CR70] Staab M, Schuldt A, Assmann T, Klein AM (2014). Tree diversity promotes predator but not omnivore ants in a subtropical Chinese forest. Ecol Entomol.

[CR71] Staab M, Bruelheide H, Durka W, Michalski S, Purschke O, Zhu CD, Klein AM (2016). Tree phylogenetic diversity promotes host–parasitoid interactions. Proc Royal Soc B.

[CR72] Staab M, Liu X, Assmann T, Bruelheide H, Buscot F, Durka W, Erfmeier A (2021). Tree phylogenetic diversity structures multitrophic communities. Funct Ecol.

[CR85] Stamp N (2003). Out of the quagmire of plant defense hypotheses. Q Rev Biol.

[CR73] Teixeira LH, Ganade G, Mazzochini GG, Kollmann J (2022). Phylogenetic distance controls plant growth during early restoration of a semi-arid riparian forest. Ecol Solut Evidence.

[CR74] Tucker CM, Davies TJ, Cadotte MW, Pearse WD (2018). On the relationship between phylogenetic diversity and trait diversity. Ecology.

[CR75] Valiente-Banuet A, Verdú M (2007). Facilitation can increase the phylogenetic diversity of plant communities. Ecol Lett.

[CR76] Van Bael SA, Brawn JD, Robinson SK (2003). Birds defend trees from herbivores in a Neotropical forest canopy. Proc Natl Acad Sci USA.

[CR77] Verdú M, Gómez-Aparicio L, Valiente-Banuet A (2012). Phylogenetic relatedness as a tool in restoration ecology: a meta-analysis. Proc Royal Soc B.

[CR78] Wang MQ, Li Y, Chesters D, Anttonen P, Bruelheide H, Chen JT, Durka W (2019). Multiple components of plant diversity loss determine herbivore phylogenetic diversity in a subtropical forest experiment. J Ecol.

[CR79] Wang MQ, Li Y, Chesters D, Bruelheide H, Ma K, Guo P-F, Zhou Q-S (2020). Host functional and phylogenetic composition rather than host diversity structure plant—herbivore networks. Mol Ecol.

[CR80] Webb CO, Ackerly DD, McPeek MA, Donoghue MJ (2002). Phylogenies and community ecology. Annu Rev Ecol Syst.

[CR81] Whelan CJ (1989). Avian foliage structure preferences for foraging and the effect of prey biomass. Anim Behav.

[CR82] Wiegand T, Uriarte M, Kraft NJB, Shen G, Wang X, He F (2017). Spatially explicit metrics of species diversity, functional diversity, and phylogenetic diversity: insights into plant community assembly processes. Annu Rev Ecol Evol.

[CR83] Wright IJ, Reich PB, Westoby M, Ackerly DD, Baruch Z, Bongers F, Cavender-Bares J (2004). The worldwide leaf economics spectrum. Nature.

[CR84] Zavala-López M, García-Lara S (2017). An improved microscale method for extraction of phenolic acids from maize. Plant Methods.

