# Supplementary material for: Influence of phylogenetic diversity of plant communities on tri-trophic interactions
Source: Oecologia. 2023 Sep 30;203(1-2):125–37. doi: 10.1007/s00442-023-05455-1 (PMC10615933; doi:10.1007/s00442-023-05455-1)
Supplement: Supplementary file 1 — Supplementary file1 (PDF 255 KB) [file 442_2023_5455_MOESM1_ESM.pdf]

Influence of phylogenetic diversity of plant communities on plant-herbivore-natural enemies interactions

Oecologia

Verónica Alavez [1,2], Rocio Santos-Gally [1,3], Manuel Gutiérrez-Aguilar [4], Ek del-Val [5] and Karina Boege [1]*

1 Instituto de Ecología, Departamento de Ecología Evolutiva, Universidad Nacional Autónoma de México C.P. 04510 México, CDMX

2 Posgrado en Ciencias Biológicas, Universidad Nacional Autónoma de México, C.P. 04510 México, CDMX

3 CONACYT-Instituto de Ecología, Departamento de Ecología Evolutiva, Universidad Nacional Autónoma de México C.P. 04510 México, CDMX

4 Departamento de Bioquímica, Facultad de Química, Universidad Nacional Autónoma de México, CP. 04510. Ciudad de México, México.

5 Instituto de Investigaciones en Ecosistemas y Sustentabilidad, Universidad Nacional Autónoma de México, C.P. 58190 Morelia, Michoacán, Mexico

*Corresponding author´s e-mail: kboege@unam.mx

Supplementary material

S1. Map of experimental plots group by blocks

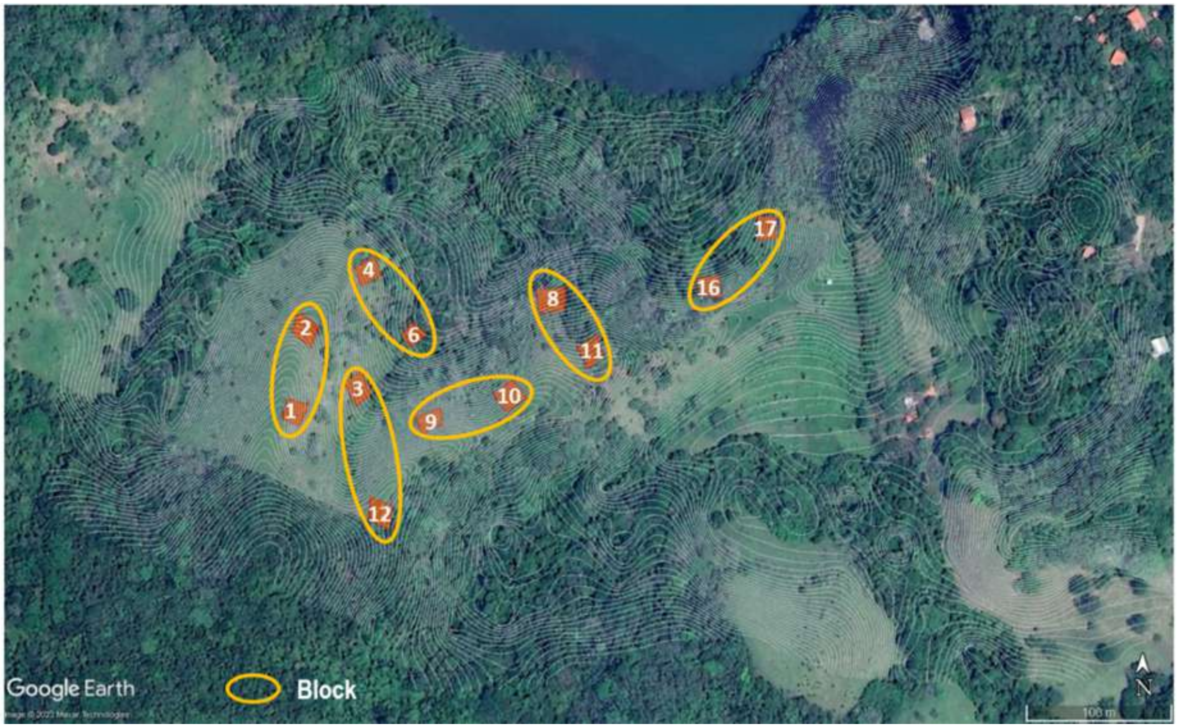

S2. Species and families in high phylogenetic diversity (HPD) and low phylogenetic diversity (LHP) plots.

Species	Family	Treatment
Rollinia jimenezii	Annonaceae	HPD
Tabernaemontana litoralis	Apocynaceae	HPD
Tabernamontana donnell-smithii	Apocynaceae	HPD
Amphitecna tuxtlensis	Bignoniaceae	HPD
Cordia stellifera	Boraginaceae	HPD
Trema micrantha	Cannabaceae	HPD
Capparis baduca	Capparaceae	HPD
Couepia polyandra	Chysobalanaceae	HPD
Diospyros digyna	Ebenaceae	HPD
Erythroxylum macrophyllum	Erythroxylaceae	HPD
Nectandra ambigens	Lauraceae	HPD
Miconia argentea	Melastomataceae	HPD
Virola guatemalensis	Myristicaceae	HPD
Coccoloba hondurensis	Polygonaceae	HPD
Psychotria limonensis	Rubiaceae	HPD
Pouteria sapota	Sapotaceae	HPD
Cestrum racemosum	Solanaceae	HPD
Vochysia guatemalensis	Vochysiaceae	HPD
Spondias mombin	Anacardiaceae	HPD/LHD
Acacia cornigera	Fabaceaea	HPD/LHD
Inga vera	Fabaceaea	HPD/LHD
Ceiba pentandra	Malvaceae	HPD/LHD
Ochroma pyramidale	Malvaceae	HPD/LHD
Heliocarpus appendiculata	Malvaceae	HPD/LHD
Cedrela odorata	Meliaceae	HPD/LHD
Guarea glabra	Meliaceae	HPD/LHD
Poulsenia armata	Moraceae	HPD/LHD
Eugenia colipensis	Myrtaceae	HPD/LHD
Zanthoxylum limoncellum	Rutaceae	HPD/LHD
Cecropia obtusifolia	Urticaceae	HPD/LHD
Spondias radlkoferi	Anacardiaceae	LHD
Sapium nitidum	Euphorbiaceae	LHD
Croton pyramidalis	Euphorbiaceae	LHD
Omphalea oleifera	Euphorbiaceae	LHD
Tetrorchidium rotundatum	Euphorbiaceae	LHD
Cojoba arborea	Fabaceaea	LHD
Hampea nutricia	Malvaceae	LHD
Bernoullia flammea	Malvaceae	LHD
Trichilia havanensis	Meliaceae	LHD
Ficus insipida	Moraceae	LHD
Ficus yoponensis	Moraceae	LHD
Trophis mexicana	Moraceae	LHD
Brosimum alicastrum	Moraceae	LHD
Pseudolmedia oxyphyllaria	Moraceae	LHD
Eugenia acapulcensis	Myrtaceae	LHD
Eugenia aeruginea	Myrtaceae	LHD
Ampelocera hottlei	Ulmaceae	LHD
Urera rzedowskii	Urticaceae	LHD

S3. Taxonomic and ecological characteristics of the study species. The ant denotes myrmecophily.

Focal species	Family		Life history
Vachellia cornigera	Fabaceae	✓	**P**
Cecropia obtusifolia	Urticaceae	✓	**P**
Inga vera	Fabaceae	✓	**T**
Ceiba pentandra	Malvaceae	✗	**P**
Eugenia colipensis	Myrtaceae	✗	**T**
Cedrela odorata	Meliaceae	✗	**P**
Zanthoxylum limoncellum	Rutaceae	✗	**T**

P: pioneer; T: tolerant

S4. Mixed model results for the growth, defenses, and leaf damage traits, evaluating the effect of treatment, life history and interaction. (*) p <0.05.

	Value	Std. Error	*DF*	t-value	*p*-value
Height growth					
Treatment	-0.11	0.12	142	-0.96	0.34
Life history	-0.14	0.13	32	-1.08	0.29
Treatment:Life history	-0.02	0.15	142	-0.15	0.88
Growth of stem diameter					
Treatment	0.03	0.11	139	0.33	0.74
Life history	-0.26	0.13	32	-1.99	0.06
Treatment:Life history	-0.19	0.14	139	-1.30	0.19
Leaf thickness					
Treatment	0.009	0.008	108	1.10	0.27
Life history	-0.03	0.01	31	-1.91	0.07
Treatment:Life history	-0.02	0.01	108	-2.18	0.03*
Specific leaf mass					
Treatment	-0.001	0.0009	97	-1.21	0.23
Life history	-0.003	0.001	25	-2.47	0.02*
Treatment:Life history	0.001	0.001	97	0.77	0.44
Free phenolic acids					
Treatment	0.13	0.16	126	0.83	0.40
Life history	-0.45	0.28	31	-1.59	0.12
Treatment:Life history	-0.43	0.21	126	-2.08	0.04*
Leaf damage					
Treatment	-0.11	0.20	118	-0.56	0.58
Life history	-0.52	0.28	32	-1.90	0.07
Treatment:Life history	0.60	0.27	118	2.20	0.03*

S5. Mixed model results implemented for clay models, assessing treatment, presence/absence of mutualistic interaction with ants, and interaction (*) $p < 0.05$.

	Estimate	Std. Error	t-value	Pr(>|z|)
Treatment	0.01	0.02	0.78	0.44
Ant interaction	0.13	0.05	2.79	0.005*
Treatment: Ant interaction	-0.12	0.05	-2.21	0.03*
